# Pharmacological differences between beta-blockers and postoperative mortality following colon cancer surgery

**DOI:** 10.1038/s41598-022-08736-6

**Published:** 2022-03-28

**Authors:** Lovisa Ekestubbe, Gary Alan Bass, Maximilian Peter Forssten, Gabriel Sjölin, Yang Cao, Peter Matthiessen, Rebecka Ahl Hulme, Shahin Mohseni

**Affiliations:** 1 School of Medical Sciences, Orebro University, 702 81 Orebro, Sweden; 2 Division of Traumatology, Surgical Critical Care and Emergency Surgery, Penn Medicine, Penn Presbyterian Medical Center, 19104 Philadelphia, USA; 3 Division of Trauma and Emergency Surgery, Department of Surgery, Orebro University Hospital, 701 85 Orebro, Sweden; 4 Clinical Epidemiology and Biostatistics, School of Medical Sciences, Orebro University, 701 82 Orebro, Sweden; 5 Division of Colorectal Surgery, Department of Surgery, Orebro University Hospital, 701 85 Orebro, Sweden; 6 Division of Trauma and Emergency Surgery, Department of Surgery, Karolinska University Hospital, Stockholm, Sweden; 7 Division of Surgery, Department of Clinical Science, Intervention and Technology, Karolinska Institutet, Stockholm, Sweden

**Keywords:** Drug development, Colorectal cancer

## Abstract

β-blocker therapy has been positively associated with improved survival in patients undergoing oncologic colorectal resection. This study investigates if the type of β-blocker used affects 90-day postoperative mortality following colon cancer surgery. The study was designed as a nationwide retrospective cohort study including all adult (≥ 18 years old) patients with ongoing β-blocker therapy who underwent elective and emergency colon cancer surgery in Sweden between January 1, 2007 and December 31, 2017. Patients were divided into four cohorts: metoprolol, atenolol, bisoprolol, and other beta-blockers. The primary outcome of interest was 90-day postoperative mortality. A Poisson regression model with robust standard errors was used, while adjusting for all clinically relevant variables, to determine the association between different β-blockers and 90-day postoperative mortality. A total of 9254 patients were included in the study. There was no clinically significant difference in crude 90-day postoperative mortality rate [n (%)] when comparing the four beta-blocker cohorts metoprolol, atenolol, bisoprolol and other beta-blockers. [97 (1.8%) vs. 28 (2.0%) vs. 29 (1.7%) vs. 11 (1.2%), *p* = 0.670]. This remained unchanged when adjusting for relevant covariates in the Poisson regression model. Compared to metoprolol, there was no statistically significant decrease in the risk of 90-day postoperative mortality with atenolol [adj. IRR (95% CI): 1.45 (0.89–2.37), *p* = 0.132], bisoprolol [adj. IRR (95% CI): 1.45 (0.89–2.37), *p* = 0.132], or other beta-blockers [adj. IRR (95% CI): 0.92 (0.46–1.85), *p* = 0.825]. In patients undergoing colon cancer surgery, the risk of 90-day postoperative mortality does not differ between the investigated types of β-adrenergic blocking agents.

## Introduction

Colorectal cancer is the second most common type of cancer in both men and women in Sweden^1^. The incidence of colorectal cancer has steadily increased in Sweden, partly due to a real increase but largely due to the fact that we are living longer and colorectal cancer affects older individuals in particular^1,2^. Although care associated with colorectal cancer has undergone major advancements over the past decades, including detection at earlier and more curable stages with screening programmes, improved diagnostic methods, and quality of treatment, colorectal cancer remains a major cause of morbidity and mortality^3–8^. It has been postulated that clinically important outcome benefits are gained by reducing the effects of surgery-induced hyperadrenergic activity in patients undergoing colon cancer surgery, through the use of β-blockade^9^.

In recent years, there has been considerable interest in the potential protective effects which preoperative beta-blockers may hold, since the immediate effect of beta-blockade is the blockage and mitigation of the sympathetic hyperactivity triggered by the trauma of surgery on several organs^9–12^. Results from two previously published studies concluded that preoperative beta-blocker use may be associated with a reduction in 30-day mortality following emergency and elective colonic cancer surgery^9^, and the same favorable effects were also seen after abdominal resection for rectal cancer^11^. In the previous large, nationwide, retrospective cohort study by Ahl et al. significantly fewer cardiovascular-related deaths were observed in patients treated with preoperative beta-blocker therapy (0.6% vs. 0.9%, *p* = 0.004), in spite of a higher prevalence of cardiovascular disease in this patient group^9^. On the other hand, in spite of these recent findings regarding the association between beta-blockers and reduced mortality, use of beta-blockers were not found to be associated with improved survival in the randomized controlled Perioperative Ischemic Evaluation (POISE) trial by Devereaux and colleagues. However, in the POISE trial the incidence of cardiovascular adverse events, such as i.e. cardiovascular death, non-fatal myocardial infarction and non-fatal cardiac arrest, were found to be reduced in their beta-blocked patient cohort^13^. Conflicting results in relation to mortality reduction similar to that of the POISE trial have also been reproduced in other studies^13–16^. This discrepancy could be attributed to the fact that these studies have tended to include several different surgical fields under the umbrella term ‘non-cardiac surgery’ i.e. orthopaedic, abdominal and vascular procedures which were included in the POISE trial. However, in the above large nationwide cohort study, only patients who underwent elective colon cancer surgery in Sweden over a 10 year period were included. The authors concluded that preoperative beta-blocker therapy is associated with considerable reductions in postoperative short-term and long-term mortality following elective colon cancer surgery^9^.

While all β-adrenoceptor blocking drugs share the common property of antagonizing the actions of the endogenous adrenergic agonists at the receptor level, there are however pharmacological differences between various agents^17–21^. For instance, in terms of pharmacokinetic variability (local anesthetic properties due to membrane stabilizing activity, K^+^-channel blocking activity, antioxidant properties, partial agonist activity, cardioselectivity) as well as pharmacodynamic differences, e.g. dose–response curves, drug-drug interactions, and in the presence of comorbidities^17–20^. This study aims to investigate and compare the effects of beta-blocker therapy on 90-day postoperative mortality risk following colon cancer surgery, based on the type of β-blocker administered. While there are pharmacological differences between the most commonly prescribed beta-blockers metoprolol, atenolol, bisoprolol and other beta-blockers, in terms of e.g. cardioselectivity and lipophilicity, it could be hypothesized that the clinically important effect is merely due to the attenuation of the hyperadrenergic response initiated due the trauma of surgery.

## Methods

Ethical approval for the execution of the study was obtained from the Swedish Ethical Review Authority, Uppsala County, Sweden (reference: 2015/454 and 2015/454/1). Patient consent was waived and approved by the ethical review authority due to the retrospective nature of the study where only unidentified personal data was obtained and no changes to care were made. All patients ≥ 18 years old with ongoing β-blocker therapy that underwent colon cancer surgery between January 1, 2007 and December 31, 2017 were considered for inclusion. Patient data were retrieved from the Swedish Colorectal Cancer Registry (SCRCR)^22^ and the Swedish National Board of Health and Welfare National Patient Registry. Data on dispensed prescriptions of beta-blockers were collected from the Swedish Prescribed Drug Register (SPDR). Patients were considered to be receiving regular beta-blocker therapy if they filled a prescription for β-blockers within the year before surgery.

Patients with β-blocker therapy were subdivided into four groups according to the type of β-blocker used prior to surgery: metoprolol, atenolol, bisoprolol, and other β-blockers. The Pearson’s chi-squared test and Fisher’s exact test were used to determine the statistical significance of differences between categorical variables. For non-normally distributed continuous variables the Kruskal–Wallis test was applied in order to calculate the statistical significance of differences between the groups. The primary outcome of interest was 90-day postoperative mortality. A Poisson regression model with robust standard errors was employed to analyze the association between the type of β-blocker and risk of 90-day postoperative mortality risk. Adjustment for potential confounding in the model was performed by including the following covariates: age, sex, American Society of Anesthesiologists (ASA) classification, Charlson Comorbidity Index (CCI), cancer stage, surgical setting (acute versus elective presentation), type of surgical resection, and operative method (open versus laparoscopic). To compensate for missing data in the covariates in the regression model, multiple imputation by chained equations was applied. For binary variables logistic regression was implemented, for nominal variables a Bayesian polytomous regression model was used, and for ordinal variables a proportional odds model was applied. Analyses were performed using the statistical programming language R^23^.

## Results

A total of 9254 patients met the specified inclusion criteria. Depicted in Tables 1 and 2 are patient demographics, comorbidity data, clinical characteristics, and surgical interventions in each beta-blocker group. Patients on bisoprolol were considered the least fit for surgery, with a larger proportion of the cohort having an ASA score of ≥ 3, followed by metoprolol, other β-blockers, and atenolol (ASA ≥ 3: 54.4% vs. 45.6% vs. 42.3% vs. 32.7%, *P* value < 0.001). In addition, patients administered bisoprolol suffered from the most comorbidities, followed by the metoprolol, other β-blocker, and atenolol cohorts (CCI ≥ 7: 34.2% vs. 28.1% vs. 24.6% vs. 19.4%, *P* value < 0.001). Most comorbidities were the most prevalent among bisoprolol users, with the exception for dementia, hemiplegia, liver disease, and cerebrovascular disease (Table 2). Furthermore, myocardial infarction, congestive heart failure, peripheral vascular disease, and chronic obstructive pulmonary disease were shown to be more prevalent in patients administered bisoprolol. Comparing the β-blocker cohorts’ characteristics, statistically significant differences were observed with regards to age, sex, ASA classification and CCI. No clinically significant differences were detected between the different beta-blocker cohorts regarding cancer stage, surgical setting, type of surgery, or operative method (Table 1).

**Table 1:** Patient demographics.

	Metoprolol N = 5252	Atenolol N = 1381	Bisoprolol N = 1696	Other N = 925	*P* value
Age, mean (SD)	75.3 (± 9.2)	74.6 (± 9.0)	75.9 (± 8.7)	73.4 (± 9.6)	< 0.001
Sex, n (%)					< 0.001
Female	2591 (49.3%)	770 (55.8%)	841 (49.6%)	458 (49.5%)	
Male	2661 (50.7%)	611 (44.2%)	855 (50.4%)	467 (50.5%)	
ASA, n (%)					< 0.001
1	275 (5.2%)	55 (4.0%)	72 (4.2%)	65 (7.0%)	
2	2433 (46.3%)	843 (61.0%)	664 (39.2%)	447 (48.3%)	
3	2166 (41.2%)	424 (30.7%)	825 (48.6%)	359 (38.8%)	
4	230 (4.4%)	27 (2.0%)	96 (5.7%)	32 (3.5%)	
5	1 (0.0%)	0 (0.0%)	1 (0.1%)	0 (0.0%)	
Missing	147 (2.8%)	32 (2.3%)	38 (2.2%)	22 (2.4%)	
CCI, n (%)					< 0.001
≤ 4	1535 (29.2%)	518 (37.5%)	407 (24.0%)	324 (35.0%)	
5–6	2242 (42.7%)	595 (43.1%)	709 (41.8%)	373 (40.3%)	
≥ 7	1475 (28.1%)	268 (19.4%)	580 (34.2%)	228 (24.6%)	
Cancer stage, n (%)					0.034
1	786 (15.0%)	208 (15.1%)	269 (15.9%)	150 (16.2%)	
2	2010 (38.3%)	525 (38.0%)	672 (39.6%)	339 (36.6%)	
3	1689 (32.2%)	415 (30.1%)	541 (31.9%)	290 (31.4%)	
4	591 (11.3%)	193 (14.0%)	161 (9.5%)	111 (12.0%)	
Missing	176 (3.4%)	40 (2.9%)	53 (3.1%)	35 (3.8%)	
Surgical setting					0.800
Elective	4403 (83.8%)	1151 (83.3%)	1431 (84.4%)	783 (84.6%)	
Acute	849 (16.2%)	230 (16.7%)	265 (15.6%)	142 (15.4%)	
Type of surgical resection , n (%)					0.083
Right hemicolectomy	2861 (54.5%)	731 (52.9%)	957 (56.4%)	513 (55.5%)	
Ileocecal resection	106 (2.0%)	21 (1.5%)	29 (1.7%)	9 (1.0%)	
Transverse colon resection	129 (2.5%)	32 (2.3%)	38 (2.2%)	23 (2.5%)	
Left hemicolectomy	559 (10.6%)	161 (11.7%)	193 (11.4%)	118 (12.8%)	
Sigmoid resection	1211 (23.1%)	343 (24.8%)	351 (20.7%)	207 (22.4%)	
Total c olectomy	196 (3.7%)	36 (2.6%)	63 (3.7%)	33 (3.6%)	
Hartmann’s operation	190 (3.6%)	57 (4.1%)	65 (3.8%)	22 (2.4%)	
Operative method					0.350
Open surgery	4826 (91.9%)	1287 (93.2%)	1556 (91.7%)	857 (92.6%)	
Laparoscopic surgery	426 (8.1%)	94 (6.8%)	140 (8.3%)	68 (7.4%)	

N, Total number of patients; ASA, American Society of Anesthesiologists; CCI, Charlson Comorbidity Index.

**Table 2:** Comorbidities.

	Metoprolol N = 5252	Atenolol N = 1381	Bisoprolol N = 1696	Other N = 925	*P* value
Myocardial infarction, n (%)	799 (15.2%)	72 (5.2%)	269 (15.9%)	94 (10.2%)	< 0.001
Congestive heart failure, n (%)	641 (12.2%)	57 (4.1%)	353 (20.8%)	101 (10.9%)	< 0.001
Peripheral vascular disease, n (%)	279 (5.3%)	44 (3.2%)	117 (6.9%)	52 (5.6%)	< 0.001
Cerebrovascular disease, n (%)	529 (10.1%)	106 (7.7%)	171 (10.1%)	67 (7.2%)	0.004
Dementia, n (%)	81 (1.5%)	8 (0.6%)	14 (0.8%)	8 (0.9%)	0.006
Chronic obstructive pulmonary disease, n (%)	330 (6.3%)	61 (4.4%)	297 (17.5%)	48 (5.2%)	< 0.001
Connective tissue disease, n (%)	168 (3.2%)	33 (2.4%)	65 (3.8%)	23 (2.5%)	0.085
Peptic ulcer disease, n (%)	266 (5.1%)	47 (3.4%)	89 (5.2%)	44 (4.8%)	0.060
Liver disease, n (%)	40 (0.8%)	6 (0.4%)	16 (0.9%)	29 (3.1%)	< 0.001
Diabetes, n (%)	918 (17.5%)	206 (14.9%)	316 (18.6%)	155 (16.8%)	0.048
Hemiplegia, n (%)	54 (1.0%)	9 (0.7%)	15 (0.9%)	4 (0.4%)	0.250
Chronic kidney disease, n (%)	180 (3.4%)	24 (1.7%)	82 (4.8%)	35 (3.8%)	< 0.001

N, Total number of patients.

There were no statistically significant differences regarding the crude 90-day postoperative mortality rate between the four cohorts of β-blocker therapy (Table 3). Table 4 shows the adjusted incidence rate ratio for 90-day postoperative mortality for the four cohorts of β-blockers. After adjustment for relevant covariates in the Poisson regression model, no difference in 90-day postoperative mortality risk could be detected. In comparison with metoprolol, there was no statistically significant change in the risk of 90-day postoperative mortality with atenolol [adj. IRR (95% CI): 1.45 (0.89–2.37), *p* = 0.132], bisoprolol [adj. IRR (95% CI): 0.83 (0.49–1.39), *p* = 0.490], or other β-blockers cohorts [adj. IRR (95% CI): 0.92 (0.46–1.85), *p* = 0.825]. However, there were statistically significant differences in risk of 90-day postoperative mortality in terms of ASA 1–2 (ref.) in comparison to ASA 3–5 [adj. IRR (95% CI): 2.62 (1.63–4.22), *p* =  < 0.001] as well as in the surgical setting when comparing elective (ref.) with emergency surgery [adj. IRR (95% CI): 4.5 (3.05–6.65), *p* =  < 0.001].

**Table 3:** Crude outcomes.

	Metoprolol N = 5252	Atenolol N = 1381	Bisoprolol N = 1696	Other N = 925	*P* value
Length of stay					0.003
Median (IQR)	8.0 (6.0–12.0)	7.0 (5.0–11.0)	7.0 (6.0–12.0)	7.0 (5.0–11.0)	
Missing	41 (0.8%)	11 (0.8%)	8 (0.5%)	8 (0.9%)	
90-day mortality, n (%)	97 (1.8%)	28 (2.0%)	29 (1.7%)	11 (1.2%)	0.670

N, Total number of patients.

**Table 4:** Incidence rate ratio for 90-day mortality after colon cancer surgery.

	IRR (95% CI)	*P* value
Type of beta-blocker		
Metoprolol	ref	
Atenolol	1.45 (0.89–2.37)	0.132
Bisoprolol	0.83 (0.49–1.39)	0.490
Other	0.92 (0.46–1.85)	0.825
Age	1.03 (1–1.05)	0.025
Sex		
Female	ref	
Male	0.79 (0.54–1.15)	0.216
ASA		
Low*	ref	
High**	2.62 (1.63–4.22)	< 0.001
CCI		
≤ 4	ref	
5–6	1.29 (0.75–2.21)	0.360
≥ 7	2.27 (1.35–3.83)	0.002
Cancer stage		
1	ref	
2	1.15 (0.55–2.4)	0.730
3	1.09 (0.51–2.31)	0.846
4	3.38 (1.61–7.06)	0.001
Surgical setting		
Elective	ref	
Acute	4.5 (3.05–6.65)	< 0.001
Type of surgical resection		
Right Hemicolectomy	ref	
Ileocecal resection	1.05 (0.4–2.73)	0.927
Transverse colon resection	1.92 (0.87–4.25)	0.107
Left Hemicolectomy	0.91 (0.46–1.77)	0.786
Sigmoid resection	1.01 (0.61–1.67)	0.977
Total c olectomy	1.17 (0.55–2.48)	0.690
Hartmann´s operation	0.53 (0.2–1.43)	0.212
Surgical Technique		
Open surgery	ref	
Laparoscopic surgery	0.37 (0.08–1.66)	0.193

*ASA 1–2.

**ASA 3–5.

Poisson regression model with robust standard errors. Model adjusted for age, sex, ASA, CCI cancer stage, surgical setting, type of surgery, and operation method.

IRR, Incidence rate ratio; ASA, American Society of Anesthesiologists; CCI, Charlson comorbidity index.

## Ethics approval

Ethical approval was obtained from The Swedish Ethical Review Authority (reference number 2015/454 and 2015/454/1). The principles of the Declaration of Helsinki and Strengthening the Reporting of Observational Studies in Epidemiology (STROBE) guidelines were complied with while conducting this study.

## Discussion

The current study demonstrates that there is no difference in the risk of 90-day postoperative mortality after colon cancer surgery based on the type of β-blocker exposure. This observation may support the hypothesis that the positive and prophylactic effects associated with β-blocker use on postoperative mortality, following both elective and emergency colon cancer surgery, is attributable to the common property of β-adrenoceptor antagonism that these agents share. Thus, although they differ from one another in their additional pharmacological properties β-blockers may be considered a class of drugs with the ability to attenuate the effects of surgery-induced hyperadrenergic state.

All β-adrenoceptor blocking drugs share the common property of antagonizing the actions of the endogenous adrenergic agonists, norepinephrine and epinephrine, at the receptor level^17,24^. In addition, there are pharmacological differences between various agents in terms of pharmacokinetic variability (local anesthetic properties due to membrane stabilizing activity, K^+^- channel blocking activity, anti-oxidant properties, partial agonist activity, cardioselectivity) as well as pharmacodynamic differences, e.g. dose–response curves, drug-drug interactions, and presence of comorbidities^17–20^. Differences in the aromatic ring structure between various types of β-blockers causes variability in lipophilic characteristics, consequently impacting pharmacokinetic properties such as hepatic extraction ratio, protein binding, volume of distribution as well as the ratio of renal versus hepatic clearance^21^.

While β-blockers typically have been classified on the basis of their cardioselectivity, the beneficial effects of β-blockade may also be examined based on the degree to which they possess other pharmacological properties such as partial agonist properties, membrane stabilization and lipophilicity. In the multicenter, prospective, observational study by Ley et al. overall 30-day mortality was 15.8% with a significantly lower mortality observed in patients administered β-blockers following hospitalization after traumatic brain injury (13.8% vs. 17.7%, *p* = 0.013)^25^. Propranolol, a non-specific β-adrenergic receptor antagonist with ability to passively cross the blood–brain barrier due to its lipophilic nature, was observed to be associated with a lower mortality compared to other beta-blockers. Propranolol, metoprolol and nebivolol^26,27^ are all considered lipophilic agents primarily eliminated by hepatic metabolism, have shorter half-lives, and greater variations in plasma concentrations^26,28^. In comparison, more hydrophilic agents e.g. nadolol, sotalol and atenolol are subjected to excretion by the kidneys and thus dosage need to be adjusted with respect to renal function^28^. Furthermore, lipophilic beta-blockers might also improve cardiac vagal activity, which has been linked to mortality, after they cross the blood–brain barrier to reach the central nervous system^26,29^.

Third-generation β-blockers, such as nebivolol and carvedilol. are agents which unlike “classic β-blockers”, exhibit vasodilating activities by antagonizing α1-adrenoreceptors and activating β3-adrenergic receptors^30,31^, Additionally, third-generation β-blockers display anti-hypertrophic, anti-apoptotic, anti-proliferative, antioxidant and angiogenic properties still under examination^31–33^. Vasodilatory β-blockers provide benefits such as reduction of peripheral vascular resistance whilst maintaining or improving cardiac output, stroke volume, and left ventricular function^34^. On the contrary, β-blockers without vasodilating properties are inclined to increase peripheral vascular resistance, decrease cardiac output and reduce left ventricular function. Consequently, vasodilating β-blockers with favorable hemodynamic effects may prove more useful in decreasing central pressure and preventing cardiovascular events. Simultaneously as the second-generation agents, such as metoprolol, bisoprolol and atenolol, exhibit higher affinity binding to β1-receptors than β2-receptors^35,36^, the degree of selectivity are not definite and ranges widely between these agents^26,37^. Bisoprolol and nebivolol possesses the highest β1-selectivity^38^, in comparison to other β-blockers used in clinical practice such as e.g. atenolol and metoprolol which are both approximately fivefold selective for beta-1 versus beta-2-receptors^39^. Moreover, the extent of selectivity may be influenced by the magnitude of the dose^37^. In lower doses, β1-selective agents antagonize cardiac β1-receptors and have less effect on vascular smooth and bronchial muscles^40^. In contrast, administered at higher dosage (e.g. > 50 mg/d of metoprolol) even β1-selective agents also block β2-receptors, which imply that cardioselective beta-blockers may result in adverse effects on patients with obstructive airways disease^26,40,41^.

In the large nationwide retrospective cohort study by Ahl et al. preoperative beta-blockade was found to be associated with considerable reductions in short- and long-term postoperative mortality following elective colon cancer surgery. The study showed a 43% risk reduction in one-year all-cause mortality (adjusted HR 0.57, 95% CI 0.52 to 0.63, p < 0.001) in beta-blocker users^9^. Similarly, Ahl et al. demonstrated, in a cohort of 11,966 patients of whom 3513 were exposed to regular beta-blockers, a strong association between beta-blocker use and improved survival and morbidity rates following abdominal resection performed for rectal cancer^11^. Furthermore, the results of this study also indicate that mortality due to cardiovascular and respiratory illness, sepsis and multiorgan failure were significantly lower among beta-blocker users. In addition, beta-blocker therapy may also provide a protective effect against the development of postoperative complications after emergency colon cancer surgery, which was earlier demonstrated in another study on the topic by Ahl and colleagues^42^. The study revealed that there was a trend toward reduced overall incidence of major postoperative complications observed in beta-blocker positive patients (adjusted IRR 0.77, 95% CI 0.59–1.00, *p* = 0.055). Kwon et al. published, in their cohort of 8431 patients undergoing elective colorectal and bariatric surgical procedures of whom 23.5% were taking beta-blockers prior to surgery, that continuation of beta-blocker therapy on the day of, and after, surgery was associated with reduced 90-day mortality and fewer cardiac events^43^. In the current study, the finding that there was no significant difference in the crude risk of 90-day postoperative mortality with atenolol, bisoprolol, other beta-blockers, compared to metoprolol, remained unchanged even after adjustment for relevant covariates in the Poisson regression model. This indicates that beta-blocker therapy, irrespective of the agent used, may decrease mortality after elective and emergency colon cancer surgery.

The present study is strengthened by the use of information obtained from an externally validated, prospectively collected database with a > 99% national inclusion coverage of colon cancer patients in Sweden as well as a more homogenous surgical population treated with standardized surgery for a single type of cancer predominantly affecting the elderly population. Meanwhile, the retrospective design does result in limitations such as unknown confounders which are not controlled for, unknown indication for issued drug prescriptions, inability to determine whether favorable effects of β-blockade are attributable to pre-, peri- and/or postoperative administration of β -blockers, and lack of information on a strict daily β-blocker compliance during and after the hospitalization. It is unfortunate that the present study did not include a patient cohort without beta-blocker therapy. However, previous studies have been published on the outcomes after colorectal surgery between beta-blocker and non-beta-blocker users. The aim of the current study was to compare the 90-day postoperative mortality after colon cancer surgery based on the type of β-blocker explaining the absence of a reference group without beta-blocker therapy. Future research may overcome these limitations, through implementation of an interventional design with application of a randomized allocation procedure to prospectively investigate the effect of different types of β-blockers on the postoperative outcome.

## Conclusion

This retrospective cohort study demonstrates that in patients undergoing elective and emergency colon cancer surgery, there was no statistically significant difference in the risk of 90-day postoperative mortality between common β-blockers.
